# Bacterial profile and drug susceptibility pattern of urinary tract infection in pregnant women at University of Gondar Teaching Hospital, Northwest Ethiopia

**DOI:** 10.1186/1756-0500-5-197

**Published:** 2012-04-25

**Authors:** Agersew Alemu, Feleke Moges, Yitayal Shiferaw, Ketema Tafess, Afework Kassu, Belay Anagaw, Abebe Agegn

**Affiliations:** 1 Department of Medical Parasitology, School of Biomedical and Laboratory Sciences, College of Medicine and Health Sciences, University of Gondar, Gondar, Ethiopia; 2 Department of Medical Microbiology, School of Biomedical and Laboratory Sciences, College of Medicine and Health Sciences, University of Gondar, Gondar, Ethiopia; 3 Department of Immunology and Molecular Biology, School of Biomedical and Laboratory Sciences, College of Medicine and Health Sciences, University of Gondar, Gondar, Ethiopia

## Abstract

**Background:**

Urinary tract infection (UTI) is a common health problem among pregnant women. Proper investigation and prompt treatment are needed to prevent serious life threatening condition and morbidity due to urinary tract infection that can occur in pregnant women. Recent report in Addis Ababa, Ethiopia indicated the prevalence of UTI in pregnant women was 11.6 % and Gram negative bacteria was the predominant isolates and showed multi drug resistance. This study aimed to assess bacterial profile that causes urinary tract infection and their antimicrobial susceptibility pattern among pregnant women visiting antenatal clinic at University of Gondar Teaching Hospital, Northwest Ethiopia.

**Methods:**

A cross-sectional study was conducted at University of Gondar Teaching Hospital from March 22 to April 30, 2011. Mid stream urine samples were collected and inoculated into Cystine Lactose Electrolyte Deficient medium (CLED). Colony counts yielding bacterial growth of 10^5^/ml of urine or more of pure isolates were regarded as significant bacteriuria for infection. Colony from CLED was sub cultured onto MacConkey agar and blood agar plates. Identification was done using cultural characteristics and a series of biochemical tests. A standard method of agar disc diffusion susceptibility testing method was used to determine susceptibility patterns of the isolates.

**Results:**

The overall prevalence of UTI in pregnant women was 10.4 %. The predominant bacterial pathogens were *Escherichia coli* 47.5 % followed by coagulase-negative staphylococci 22.5 %, *Staphylococcus aureus* 10 %, and *Klebsiella pneumoniae* 10 %. Gram negative isolates were resulted low susceptibility to co-trimoxazole (51.9 %) and tetracycline (40.7 %) whereas Gram positive showed susceptibility to ceftriaxon (84.6 %) and amoxicillin–clavulanic acid (92.3 %). Multiple drug resistance (resistance to two or more drugs) was observed in 95 % of the isolates.

**Conclusion:**

Significant bacteriuria was observed in asymptomatic pregnant women. Periodic studies are recommended to check the outcome of asymptomatic bacteriuria and also monitor any changes in the susceptibility patterns of urinary tract pathogens in pregnant women.

## Background

In the female human subject, the urinary tract has an important relationship with the reproductive organs because of its proximity. In the non-pregnant state, the uterus lies just behind and partly over the bladder while in the pregnant state the enlarging uterus affects all the tissues of the urinary tract at various times [1]. Urinary Tract Infection (UTI) has become the most common hospital-acquired infection, accounting for as many as 35 % of nosocomial infections, and it is the second most common cause of bacteraemia in hospitalized patients [2]. Urinary tract infection accounts for a significant part of the work load in clinical microbiology laboratories and enteric bacteria remained the most frequent cause of UTI, although the distribution of pathogens that cause UTI is changing [2].

Urinary tract infection is a common health problem among pregnant women [1]. This usually begins in week 6 and peaks during weeks 22 to 24 of pregnancy due to a number of factors including urethral dilatation, increased bladder volume and decreased bladder tone, along with decreased urethral tone which contributes to increased urinary stasis and ureterovesical reflux and up to 70 % of pregnant women develop glycosuria, which encourages bacterial growth in the urine [3].

UTI may manifest as asymptomatic bacteriuria (ASB) or symptomatic bacteriuria (SB). The prevalence of asymptomatic UTI has been previously reported to be 2 % to 13 % in pregnant women [4-6] compared with that of symptomatic UTI which accounts 1–18 % during pregnancy [7]. The highest incidence of urinary tract infection occurs in the child bearing age and this has been linked to sexual activity and aging [8]. The predominant organisms that cause UTIs during pregnancy are *E. coli* which account for 80 %–90 % of infection [9,10].

Treatment of asymptomatic bacteriuria offers no benefit for most healthy an elderly or adult non pregnant women [11]. However, screening and treatment of asymptomatic bacteriuria should be performed in pregnant women [12]. Treatment of UTI in pregnancy is of paramount importance for mother and child. Given the close association between ASB and obvious UTI, screening and treatment of ASB in pregnancy may also help to reduce adverse outcome for the child such as pre-term labour and low birth weight [12]. However, Antibiotic resistance of urinary tract pathogens has been known to increase worldwide, especially to commonly used antimicrobials [13] and pattern of antibiotic resistance in a wide variety of pathogenic organisms may vary over short periods and depend on site of isolation and different environmental conditions [14].

In many hospitals in developing countries including Ethiopia, routine urine culture test is not carried out for antenatal patients instead many clinicians choose for the strip urinalysis method for assessing urine in pregnant women. The true picture of such urine specimen cannot be fully assessed as the strip cannot quantify the extent of infection in such a patient as well as provide antimicrobial therapy which is usually seen in the case of culture test. Antibiotics are usually given empirically before the laboratory results of urine culture are available. To ensure appropriate therapy, current knowledge of the organism that causes UTIs and their antibiotic susceptibility pattern is mandatory. Therefore, periodic evaluation of antibacterial activity is needed to update information. Therefore, to readdress this situation, this study was aimed to isolate and identify the bacterial etiologic agents, including their antibiotic susceptibility pattern isolated from pregnant women with urinary tract infection visiting University of Gondar Teaching Hospital, Northwest Ethiopia.

## Methods

### Study design and area

A cross sectional study was conducted at the University of Gondar Teaching Hospital from March 22 to April 30, 2011. Gondar is located 738 Kilo meter far from Addis Ababa (the capital city) to the Northwest Ethiopia. The University Hospital is one of the biggest tertiary level referral and teaching hospitals in the region that provides services to over 5 million inhabitants in the Northwest Ethiopia.

### Study populations

The study population was those pregnant women attending antenatal clinic (ANC) at University of Gondar Teaching Hospital during the study period and who did not initiate of antibiotics therapy during the last two weeks and during data collection.

### Sample size and sampling techniques

#### Sample size determination

A single proportion formula was used to calculate the sample size, n = Z^2^ p (1-p) / d^2^. Where: Z = Z score for 95 % confidence interval = 1.96, p = prevalence, d = tolerable error =5 %. Prevalence of 50 % was considered since the prevalence of UTI in pregnant women is not determined in the study area. There by n = (1.96)^2^ 0.5(1–0.5)/ (0.05)^2^ =385, giving the final sample size of 385.

### Sampling methods

The list of all pregnant women was obtained from antenatal clinic (ANC). Study participants were selected using random systematic sampling technique taking every n + 1 starting from serial number one.

#### *Inclusion criteria*

All pregnant women without current antibiotics therapy and welling to participate were included.

#### *Exclusion criteria*

Pregnant women with current antibiotics therapy were excluded from the study.

### Sample collection and processing

#### *Sample collection*

Three hundred eighty five early morning 5 ml of mid-stream urine specimens were collected using lick proof re-usable plastic containers. All of the specimens were analyzed within an hour of collection. Socio-demographic variables (Age, Sex, Marital status, Education level, Occupation, Residence and other relevant clinical data such as Parity, Gravidity, Trimester, History of catheterization, History of UTI) were obtained using a pre-designed questionnaire.

### Bacteriological investigation

#### *Culture*

Using calibrated wire loop (0.001 ml) samples were inoculated in to Cystine Lactose Electrolyte Deficient medium (CLED). After overnight incubation at 37°C for 24–48 hours colonies were counted to check significant growth. Colony counts yielding bacterial growth of 10^5^/ml of urine were regarded as significant for bacteriuria. Colonies from CLED were sub cultured into MacConkey agar and blood agar plates (BAP) (Oxoid, LTD) and incubated at 37°C for 24–48 hours. Identification of bacteria was done using colony characteristics, gram reaction of the organisms and biochemical test following standard procedure [15]. All procedures were conducted in University of Gondar Teaching Hospital Microbiology Laboratory by the same senior laboratory technologist in all the time to avoid professional biases.

#### *Antimicrobial susceptibility testing*

Susceptibility testing was performed on all isolates according to the criteria of National Committee for Clinical Laboratory Standards (NCCLs) [16].The drugs that were tested include amoxicillin (10 μg), amoxicillin-clavulinic acid (20 μg), ampicillin (30 μg), ciprofloxacin (5 μg), Norfloxacin (10 μg), gentamicin (10 μg), tetracycline (30 μg), co-trimoxazole (25 μg) chloroamphenicol (30 μg) and ceftriaxon (10 μg).

### Quality control

Culture media were tested for sterility and performance. Standard strains of *E. coli* ATCC 25922 and *S. aureus* ATCC 25923 were used during culture and antimicrobial susceptibility testing.

### Data management and analysis

Data were entered into a database designed using MS Excel spreadsheet and analyzed using SPSS statistical software package (version 16). Study findings were explained in words and tables. Proportions for categorical variables were compared using chi-square test. In all cases *P-value* less than 0.05 was taken as statistically significant.

### Ethical considerations

An ethical approval was obtained from ethical clearance committee of College of Medicine and Health Sciences, University of Gondar. Informed written consent was obtained from the study participants. Participants were given a full right to continue or withdraw from the study. For each confirmed infection case, the responsible clinician of the participant was informed and treatment was started as per the culture result and drug susceptibility pattern. Information obtained at each course of the study was kept confidential. Those identified positive for bacteriuria during the study period were referred to attending physicians and treated by appropriate drugs in line with the national guidelines for treatment of pregnant women.

## Results

### Sociodemographic characteristics

A total of 385 pregnant women were enrolled in this study with the age ranges of 17–45 years with the mean (standard deviation) of 26 (+5.3) years. Among the study subjects, 383 (99.5 %) were married. Two hundred ten (54.5 %) had educational level of secondary and above and 338(87.8 %) were urban dwellers. Based on their parity, 144 (37.4 %), 83 (21.6 %), and 158 (41 %) were nulliparous, monoparous, multiparous, respectively. Whereas, 168 (43.7 %), 166 (43.1 %) and 51 (13.2 %) of study subjects were in the 3^rd^, 2^nd^ and 1^st^ trimester of pregnancy. Thirteen (3.4 %) and 47 (12.2 %) of study subjects had history of previous catheterization and urinary tract infection, respectively (Table 1).

**Table 1:** Prevalence of UTI and demographic characteristic of study population (N = 385) at Gondar University Hospital, March 22 to April 30, 2011

**Characteristics**	**No. Tested (%)**	**No. negative (%)**	**No. Positive (%)**	**Chi-square (x^2^)**	**P-value**
**Age(years)**					
16-20	71(18.4)	63(88.7)	8(11.3)		
21-25	128(33.3)	115(89.8)	13(10.2)		
26-30	132(34.3)	119(90.2)	13(9.8)	1.840	0 .871
31-35	32(8.3)	29(90.6)	3(9.4)		
36-40	19(4.9)	17(89.5)	2(10.5)		
41-45	3(0.8)	2(66.7)	1(33.3)		
**Address**					
Urban	338(87.8)	303(89.6)	35(10.4)	0.004	0.952
Rural	47(12.2)	42(89.4)	5(10.6)		
**Marital status**					
Married	383(99.5)	343(89.6)	40(10.4)	0.233	0.629
Other	2(.5)	2(100)	0(0)		
**Educational status**					
Illiterate	67(17.4)	64(95.6)	3(4.5)		
Primary(1–8)	108(28.1)	96(88.9)	12(11.1)	3.503	0.320
Secondary(9–12)	140(36.4)	122(87.1)	18(12.9)		
Higher ≫ 12	70(18.1)	63(90)	7(10)		
**Symptom of UTI**					
Yes	85(22.1)	72(84.7)	13(15.3)	2.818	0.093
No	300(77.9)	273(91)	27(9)		
**Gravidity**					
1-3	324(84.2)	291(89.8)	33(10.2)		
4-6	47(12.2)	42(89.4)	5(10.6)	0. 482	0. .923
7-9	13(3.4)	11(84.6)	2(15.4)		
≫9	1(.2)	1(100)	0(0)		
**Parity**					
Nullipara	144(37.4)	131(91)	13(9)		
One	83(21.6)	75(99.4)	8(9.6)	0.791	0.673
Multipara	158(41)	139(88)	19(12)		
**Trimester**					
1^st^	51(13.2)	50(98)	1(2)		
2^nd^	166(43.1)	148(89.2)	18(10.8)	4.732	0.094
3^rd^	168(43.6)	147(87.5)	21(12.5)		
**History of catheterization**					
Yes	13(3.4)	8(61.5)	5(38.4)	11.388	0.001
No	372(96.6)	337(90.6)	35(9.4		
**History of UTI**					
Yes	47(12.2)	34(72.3)	13(6.4)	17.150	0.001
No	338(87.8)	311(92)	27(8)		

### Prevalence of urinary tract infection

The overall prevalence of UTI was 10.4 %. Of all considered variables only history of catheterization and previous history of UTI were significantly associated with UTI. Out of 40 pregnant women who had significant bacteriuria, 13 (32.5 %) had a history of UTI in their pregnancy and received antibiotic for that UTI (P =0.001), and 5 (12.5 %) had a history of catheterization (P = 0.001). There was no association between maternal age, address, parity, gravidity, trimester, occupation, marital status and education with bacteriuria (Table1).

### Isolation and identification of bacterial uropathogens

Of all the bacteria isolated (n = 40), Gram-negative bacteria were more prevalent 27 (67.5 %) than Gram-positive bacteria 13 (32.5 %). The most commonly isolated bacteria were *E. coli* 19 (47.5 %), followed by coagulase negative Staphylococci 9 (22.5 %), *S. aureus* and *K. pneumoniae* 4 (10 %) each. *Enterobacter aerogenes* and *Morganella morgani* accounts, 2 (5 %) each (Table 2).

**Table 2:** Frequency of bacterial uropathogens isolated from pregnant women (N = 40) at Gondar University Hospital, March 22 to April 30, 2011

**Bacterial isolates**	**Total (%)**
*Escherichia coli*	19 (47.5)
*Enterobacter aerogenes*	2 (5)
*Klebsiella pneumoniae*	4 (10)
*Morganella morgani*	2 (5)
CNS	9 (22.5)
*Staphylococcus aureus*	4 (10)
**Total**	**40(100)**

*CNS = coagulase negative staphylococcus.

### Antimicrobial susceptibility pattern of bacterial uropathogens

The result of antimicrobial susceptibility pattern of the isolate is shown on Tables 3 and 4 below. Rates of susceptibility of Gram-negatives range from 0 % - 100 %. All isolates were sensitive to chloramphenicol 27 (100 %). Most of Gram-negative isolates were sensitive to ceftriaxon 26 (96.3 %), ciprofloxacin 26 (96.3 %), norfloxacin 25 (92.6 %), gentamicin 25 (92.6 %), amoxicillin-clavulanic acid 16 (59.3 %), co-trimoxazole 14 (51.9 %), and tetracycline 11 (40.7 %). However, all solates of gram-negetive bacteria were were resistance to ampicillin, and amoxacillin 27 (100 %). Among the Gram negatives, the predominant isolate was *E. coli, 19* (70.4 % of the Gram-negatives, 47.5 % of all isolate) demonstrated high level of resistance to ampicillin and amoxacillin 19 (100 %) followed by tetracycline 10 (52.6 %). All isolates of *E.coli* were sensitive to ceftriaxon, chloramphenicol, ciprofloxacin and norfloxacin 19 (100 %), followed by gentamicin 18 (94.7 %) (Table 3).

**Table 3:** Antimicrobial susceptibility pattern of Gram-negative bacteria isolated from urine culture of pregnant women (N = 27) at Gondar University Hospital, March 22 to April 30, 2011

**Bacterialisolate**	**Total No.**	**Pattern**	**Antimicrobial agents tested**									
			**AMP**	**AMOX**	**CRO**	**CAF**	**CIP**	**CN**	**NOR**	**SXT**	**TTC**	**AMC**
			**No.(%)**	**No. (%)**	**No.(%)**	**No.(%)**	**No. (%)**	**No. (%)**	**No. (%)**	**No.(%)**	**No. (%)**	**No (%)**
***E. coli***	19	S	0(0)	0(0)	19(100)	19(100)	19(100)	18(94.7)	19(100)	14(73.7)	9(47.4)	**12(63.2)**
		R	19(100)	19(100)	0(0)	0(0)	0((0)	1(5.3)	0(0)	5(26.3)	10(52.6)	**7(36.8)**
***Enterobacter aerogenes***	2	S	0(0)	0(0)	2(100)	2(100)	2(100)	2(100)	2(100)	0(0)	1(50)	**1(50)**
		R	2(100)	2(100)	0(0)	0(0)	0(0)	0(0)	0(0)	2(100)	1(50)	**1(50)**
***Klebsiela pneumoniae***	4	S	0(0)	0(0)	3(75)	4(100)	3(75)	3(75)	2(50)	0(0)	0(0)	**2(50)**
		R	4(100)	4(100)	1(25)	0(0)	1(25)	1(25)	2(50)	4(100)	4(100)	**2(50)**
***Morganella morgani***	2	s	0(0)	0(0)	2(100)	2(100)	2(100)	2(100)	2(100)	0(0)	0(0)	**1(50)**
		R	2(100)	2(100)	0(0)	0(0)	0(0)	0(0)	0(0)	2(100)	2(100)	**1(50)**
**Total**	27	S	0(0)	0(0)	26(96.3)	27 (100)	26(96.3)	25(92.6)	25(92.6)	14(51.9)	11(40.7)	**16(59.3)**
		**R**	**27(100)**	**27(100)**	**1(3.7)**	**0(0)**	**1(3.7)**	**2(7.4)**	**2(7.4)**	**13(48.1)**	**16(59.3)**	**11(40.7)**

**AMP** = ampicillin **CAF** = chloramphenicol **NOR** = norfloxacin.

**AMOX** = amoxacillin **CIP** = ciprofloxacin SXT = co-trimoxazole.

**CRO** = ceftriaxon **CN** = gentamicin TTC = tetracycline.

AMC = amoxicillin-clavulinic acid.

**Table 4:** Antimicrobial susceptibility pattern of Gram-positive bacteria isolated from urine culture of pregnant women (N = 13) at Gondar University Hospital, March 22 to April 30, 2011

**Bacterial isolate**	**Total No.**	**Pattern**	**Antimicrobial agents tested**									
			**AMP**	**AMOX**	**CRO**	**CAF**	**CIP**	**CN**	**NOR**	**SXT**	**TTC**	**AMC**
			**No.(%)**	**No. (%)**	**No.(%)**	**No.(%)**	**No.(%)**	**No. (%)**	**No.(%)**	**No.(%)**	**No. (%)**	**No (%)**
**CNS**	9	S	1(11.1)	3(33.3)	7(77.8)	3(33.3)	5(55.6)	8(88.9)	5(55.6)	2(22.2)	2(22.2)	8(88.9)
		R	8(88.9)	6(66.7)	2(22.2)	6(66.7)	4(44.4)	1(11.1)	4(44.4)	7(77.8)	7(77.8)	1(11.1)
***S.aureus***	4	S	1(25)	2(50)	4(100)	2(50)	3(75)	4(100)	2(50)	2(50)	1(25)	4(100)
		R	3(75)	2(50)	0(0)	2(50)	1(25)	0(0)	2(50)	2(50)	3(75)	0(0)
Total	13	S	2(15.4)	5(38.5)	11(84.6)	5(38.5)	8(61,5)	12(92.3)	7(53.8)	4(30.8)	3(23.1)	12(92.3)
		R	11(84.6)	8(61.5)	2(15.4)	8(61.5)	5(38.5)	1(7.7)	6(46.2)	9(69.2)	10(76.9)	1(7.7)

AMP = ampicillin CAF = chloramphinicol NOR = norfloxacin.

AMOX = amoxacillin CIP = ciprofloxacin SXT = co-trimoxazole.

**CRO** = ceftriaxon CN = gentamicin TTC = tetracycline.

AMC = amoxicilin + clavulinic acid.

Majority of Gram positives were resistant to most of the antibiotics tested than the Gram negatives. As indicated on Table 4, the rates of susceptibility of Gram- positives range from 20 %-90 %. Among the Gram-positives, 11 (84.6 %), 12 (92.3 %) and 12 (92.3 %) of the isolates were sensitive to ceftriaxon, gentamicin and amoxicillin-clavulanic acid respectively. Coagulase negative staphylococci*,* which were the predominant isolates from Gram-positives 9 (69.2 %), were resistance to most of the antibiotics tested. Their resistance patterns of the isolates were found to be 8 (88.9 %) for ampicillin, 7 (77.9 %) for co-trimoxazol and tetracycline, and 6 (66.7 %) for amoxacillin and chloramphenicol. Gentamicin and amoxicillin –clavulanic were found to be effective against 8 (88.9 %) coagulase negative staphylococci (Table 4).

### Multiple drug resistance patterns of the isolates

Among the total isolates (n = 40) multi drug resistance (MDR = resistance in ≥ 2 drugs) were recorded in 38 (95 %) of all bacterial uropathogens. All isolates of Gram negative bacteria and 84.6 % of Gram positive bacteria showed resistance for two or more drugs (Table 5).

**Table 5:** Multi drug resistance pattern of bacterial isolates of pregnant women (N = 40) at Gondar University Hospital, March 22 to April 30, 2011

		**Antimicrobial pattern**					
**Bacterial isolate**	**Total (%)**	**R0**	**R1**	**R2**	**R3**	**R4**	**≥R5**
**Gram negative**	**27(67.5)**	0(0)	0(0)	**8(29.6)**	**3(11.1)**	**6(22.2)**	**10(37)**
*Klebsiella species*	4(14.8)	0(0)	0(0)	0(0)	0(0)	0(0)	4(100)
*Escherichia coli*	19(70.4)	0(0)	0(0)	8(42.1)	2(10.5)	5(26.3)	4(21.2)
*Enterobacter aerogenes*	2(7.4)	0(0)	0(0)	0(0)	1(50)	0(0)	1(50)
*Morganella morgani*	2(7.4)	0(0)	0(0)	0(0)	0(0)	1(50)	1(50)
**Gram positive**	**13(32.5)**	**1(7.9)**	**1(7.9)**	0(0)	**2(15.4)**	**3(23)**	**6(46.2)**
*Staphylococcus aureus*	4(30.8	1(25)	0(0)	0(0)	0(0)	1(25)	2(50)
CNS	9(69.2)	0(0)	1(11.1)	0(0)	2(22.2)	2(22.2)	4(44.4)
**Total**	**40(100)**	**1(2.5)**	**1(2.5)**	**8(20)**	**5(12.5)**	**9(22.5)**	**16(40)**

**R0**- No antibiotic resistance, **R1**- Resistance to one, **R2**-Resistance to two , **R3**-Resistance to three, **R4**- Resistance to four, **≥ R5**-resistance to five and more drugs.

## Discussion

The overall prevalence of urinary tract infection in pregnant women in this study was 10.4 %. This is comparable to the prevalence of UTI reported in Addis Ababa, Ethiopia (11.6 %) [17], but lower than study in Northern Tanzania (16.4 %) [18], Mwanza, North-western Tanzania (14.6 %) [19], and Khartoum North Hospital, Sudan (14.0 %) [20].This variation may be explained by the fact that differences in the environment, social habits of the community, the standard of personal hygiene and education.

There was no association between maternal age, address, parity, gravidity, occupation, marital status and education with bacteriuria in this study. This was in agreement with studies in Tanzania [19] and Sudan [20]. Indifferent from our result other study showed that maternal age and parity have been previously observed as risk factors for UTI among pregnant women [21]. However, Closer analysis of the published literature reveals that the age and parity effects are poorly characterized. For example, some studies showed that the prevalence of UTI increased with age [22], while others found it more with a younger age group [23].

The prevalence of urinary tract infection in pregnant women with previous history of urinary tract infection was significantly higher than those without previous history (p = 0.001). This result agrees with research work in Pakistan [24]. This might be due to presence of resistance strains from those who had previous history of UTI. Our study also showed the prevalence of urinary tract infection in pregnant women with previous history of catheterization was significantly higher than those without history of previous catheterization (P = 0.001). This finding agrees with previous report in Gondar [25].This could be due to long duration of catheterization, frequent catheterization or contamination during inserting catheters.

Gram-negative bacterial isolate were more prevalent (67.5 %) than Gram-positive bacterial isolates (32.5 %). Comparable rate of isolation of Gram-negative and Gram-positive bacteria, 60 % and 40 %, were reported in Tikur Anbessa Specialized Hospital Addis Ababa, Ethiopia [17] and 61.9 % and 38.1 % in Tanzania [26]. This could be due to the presence of unique structure in Gram negative bacteria which help for attachment to the uroepithelial cells and prevent bacteria from urinary lavage, allowing for multiplication and tissue invasion – resulting in invasive infection and pyelonephritis in pregnancy [9].

*E. coli* was the most predominant pathogen with over all isolation rates of 47.5 %. Comparable findings have been reported in Yemen, 41.5 % [27], Nigeria, 42.1 % [28], Khartoum North Hospital, Sudan 42.4 % [20], and Tikur Anbessa Specialized Hospital Addis Ababa, Ethiopia 44 % [17]. *E. coli* is the most common microorganism in the vaginal and rectal area [29]. Because of anatomical and functional changes and difficulty of maintaining personal hygiene during pregnancy, may increase the risk of acquiring UTI from *E. coli*. Coagulase-negative staphylococci (CNS) were the second dominant pathogen with overall isolation rate of 22.5 %. Comparable findings also reported from Tikur Anbessa Specialized Hospital Addis Ababa, Ethiopia 16 % [17], and Tanzania 16.7 % [26].

Antimicrobial resistance among uropathogens to the commonly used antibiotics become increasing that make clinicians with very few choices of drugs for the treatment of urinary tract infection [17,30]. In this study, susceptibility pattern of Gram-negative bacteria showed that most of the isolates were sensitive to chloramphenicol (100 %), ceftriaxon (96.3 %), ciprofloxacin (96.3 %), gentamicin (92.6), norfloxacin (92.6), amoxicillin-clavulanic acid (59.3 %), co-trimoxazole (51.9 %), and tetracycline (40.7 %). In contrary, a study in Tikur Anbessa Specialized Hospital Addis Ababa, Ethiopia [17] indicated that the susceptibility pattern for Gram-negative bacteria were amoxicillin-clavulanic acid (70 %), chloramphenicol (83.3 %), gentamicin (93.3 %) and co-trimoxazole (73.3 %). The easy availability and indiscriminate use of commonly used drugs such as co-trimoxazole and tetracycline may lead to an increase to resistance.

Among Gram-positive bacteria tested for the available drugs, some of the isolates showed susceptibility of (92.3 %), (92.3 %) and (84.6 %), to amoxicillin–clavulanic acid, gentamicin, and ceftriaxon, respectively. This is not in line with the report in Tikur Anbessa Specialized Hospital Addis Ababa, Ethiopia [17] which reported sensitivity of 100 % to amoxicillin-clavulanic acid and 85 % to gentamicin. In this study the effectiveness of amoxicillin-clavulanic acid to Gram-positive bacteria is reduced from comparable study. This may be explained as amoxicillin-clavulanic acid may be used for empiric therapy.

Multi drug resistance (MDR = resistance in ≥ 2 drugs) was seen in 95 % of the isolated bacterial uropathogens. A lower finding was reported in Tikur Anbessa Specialized Hospital Addis Ababa, Ethiopia 74 % [17].This indicates that multi drug resistance was found to be very high to the commonly used antibiotics. Antibiotic resistance has been recognized as the consequence of antibiotic use and abuse [31]. Therefore, the reasons for this alarming phenomenon might be inappropriate and incorrect administration of antimicrobial agents in empiric therapies and lack of appropriate infection control strategies, which can cause a shift to increase prevalence of resistant organism in the community.

## Conclusion

In the current study, the overall prevalence of asymptomatic UTI was 10.4 %. UTI prevalence was positively associated with previous history of catheterization and UTI. *E. coli* were the most predominant organisms followed by coagulase negative staphylococci and most of the bacterial isolates were sensitive to gentamicin, ceftriaxon, ciprofloxacin, norfloxacin, and chloroamphenicol. A large number of the isolates were resistant to ampicillin, amoxacillin, tetracycline, and co-trimoxazole. Multi-drug resistance bacteria were common. Periodic and continuous follow up are mandatory to reduce the consequence of asymptomatic bacteriuria and multi-drug resistance bacteria in pregnancy.

## Authors’ contributions

AA was the primary researcher, conceived the study, designed, participated in data collection, conducted data analysis, drafted and finalized the manuscript for publication. FM, KT, YS and AK assisted in data collection and reviewed the initial and final drafts of the manuscript. AA, FM, YS and BA interpreted the results, and reviewed the initial and final drafts of the manuscript. All authors read and approved the final manuscript.

## Competing interests

The authors declare that they have no competing interests.
